# Does the reform of equalization of basic public services affect residents' sense of safety?—A case from China

**DOI:** 10.3389/fpubh.2026.1802557

**Published:** 2026-04-09

**Authors:** Zhengqi Han

**Affiliations:** Policing School, People's Public Security University of China, Beijing, China

**Keywords:** difference-in-differences method, equalization of basic public services, people's well-being, residents' sense of safety, social security reform

## Abstract

**Background:**

The equalization of basic public services has emerged as a paramount objective in global social policy reform, particularly within developing nations. By enhancing the quality and accessibility of critical sectors—including healthcare, elderly care, and unemployment insurance—this agenda serves as a cornerstone for safeguarding social stability and fostering inclusive development. While the international community increasingly acknowledges the nexus between equitable service provision and residents' perceived safety, the existing literature remains deficient in two key areas: first, a systematic investigation of the causal mechanisms linking these variables; and second, robust empirical evidence from emerging economies like China. Addressing these gaps is crucial for deriving generalizable insights to inform global policy practices.

**Objective:**

This study examines the influence of basic public service equalization reforms on residents' sense of safety in developing countries. It also seeks to clarify the underlying mechanisms of this impact.

**Methods and samples:**

Drawing on longitudinal data from the Chinese Social Survey (CSS) covering the period from 2006 to 2021, this study leverages China's social security reforms aimed at equalizing urban-rural basic public services as a quasi-natural experiment. Employing a difference-in-differences (DID) estimation strategy, we empirically investigate the causal mechanisms through which these policy interventions influence residents' sense of safety.

**Results:**

The findings are as follows: (1) Basic public service equalization policies positively enhance residents' sense of safety. (2) Basic public service equalization exerts a positive impact on residents' sense of safety through three mediating variables: social equity, social support, and social trust. (3) The implementation of basic public service policies in provincial-level units exhibits spatial spillover effects. It is stronger for rural residents than urban residents, for residents in high-unemployment areas than those in low-unemployment areas, and for residents in regions with low per capita GDP than those in regions with high per capita GDP. (4) The short-term effect of basic public service equalization on residents' sense of safety is more prominent than its long-term effect.

**Conclusion:**

This study enriches the studies on the relationship between social security policy and residents‘ sense of safety. It provides empirical evidence for optimizing social security policies and offers insights for governments to enhance citizens' sense of residential safety. Additionally, it enriches the theoretical framework of cross-national policy evaluation and subjective sense of safety research. Beyond China-specific insights, the findings provide empirical evidence for developing countries worldwide to optimize their basic public service guarantee systems, particularly in balancing regional and group differences. Furthermore, the identified mechanisms have actionable implications for advancing the global agenda of fostering social cohesion and promoting inclusive social governance through public service reforms.

## 1 Introduction

To meet the livelihood needs of people in various countries, basic public service systems have become a key component of policy reforms worldwide. The construction of basic public service equalization primarily involves establishing comprehensive social security systems in high-welfare countries. Second, it entails adopting central or local fiscal policies to ensure equal access to medical services (1). The reform of social security affects the happiness of a country's people (2) and sense of fairness (3), so as to reduce social conflicts caused by unemployment and poverty (4), Creating a stable and orderly social environment, which in turn affects the safety perception of individuals in a country.

Among developing countries, represented by China, basic public service equalization is adopted to boost domestic demand, which enhance residents' happiness, sense of gain, and sense of safety. Meanwhile, China's public service policy reforms have faced problems in recent years. These include gaps in urban-rural services and barriers to cross-provincial accessibility. Whether such policies positively affect residents' sense of safety remains to be discussed (5). Therefore, China is a typical sample for exploring whether improving basic public services could enhance residents' sense of safety.

This study employs the difference-in-differences (DID) method. It uses data from the Chinese Social Survey (CSS) to conduct multi-period baseline regressions. Building on existing literature, this study explores two key questions: whether basic public service equalization affects residents' sense of safety, and through which mechanisms such effects operate. Heterogeneity analysis is conducted to examine how urban-rural basic public service equalization affects residents' sense of safety, with different factors influencing this effect. Additionally, given spillover effects across adjacent provinces and municipalities, this study employs the triple difference (DDD) method for regression analysis to further explore the mechanism underlying sense of safety under the combined effects of time.

## 2 Policy context and research progress

### 2.1 Policy context

Basic public service equalization had become a significant direction of social policy reform worldwide. In this process, fiscal investment and regional economic development are considered key factors. They shape the supply of social security services and increase the degree of equalization between urban and rural areas. Over time, the concept of public service provision has undergone significant changes. The traditional dual framework—where governments provide public goods and markets provide private goods—has gradually evolved. Modern governments now take on broader responsibilities. They provide basic education, basic healthcare, affordable housing, and other essential services.

China attaches great importance to people's well-being. Over the past 15 years, it has steadily implemented policies to equalize basic public services. In 2011, China established a nationwide health insurance system. In 2013, the government introduced a series of new social security policies in a centralized manner and clarified the requirements for reforming the basic public service system [(6), p. 24]. In 2015, China established the occupational pension system for employees in government agencies and public institutions. In 2018, it introduced a central adjustment fund for provincial pension insurance funds. In 2021, the Central Economic Work Conference emphasized that achieving shared prosperity requires making every effort while keeping within the limits of available resources. It also required the construction of a sound institutional framework for public service policies. The conference called for deploying customized basic public services in the core livelihood fields of public priority, including education, healthcare, elderly care services, and affordable housing provision. Since 2023, China has issued a series of policies to advance the equalization of basic public services. These policies aim to continuously enhance the balance, convenience, and accessibility of basic public services (7). The emphasis on improving people's happiness, sense of gain, and sense of safety has become a key guiding principle of China's national development. This orientation makes China a representative case for studying how basic public service equalization reforms enhance residents' sense of safety.

### 2.2 Research progress

In research on the equalization of basic public services, it begins with studies of the supply of social security services and involves the systematic work of the national government to improve welfare levels. In terms of the fairness aspect of the implementation of basic public services, the literature analyzed its impact on residents and racial groups from the perspective of a justice vision and conducted research using policy and institutional texts (8). Research on basic public service equalization has mainly focused on the effects of regional fiscal policies within specific countries. However, when taken together, existing research remains insufficient in systematically unpacking the underlying mechanisms through which equalization-oriented policies shape relevant social processes and outcomes. More importantly, empirical and theoretical investigations that explicitly conceptualize and operationalize such equalization policies as core independent variables remain severely limited, resulting in a notable gap in understanding how policy interventions independently drive variations in social indicators. Therefore, this study aims to examine the influence of basic public service equalization on residents' sense of safety. It employs a policy-fixed effects model combined with the difference-in-differences (DID) method. Moreover, this study uses residents' participation in medical insurance as an indicator of policy change. It then explores the mechanisms through which these policy shifts may influence residents' sense of safety.

In terms of safety, current research defines it primarily in two distinct perspectives. One perspective views it as a fear of crime, while the other considers it a subjective evaluation of the safety situation. The previous group of researchers studied safety through the concept of fear of crime [(9), pp. 40–43]. Another group of researchers defines safety as “the subjective feeling and residents' perception regarding the social security situation,” that is, safety refers to residents' subjective perception and evaluation of the social security situation (10). The research related to the measurement of residents' sense of safety in the positive direction can be classified into three categories: individual factors, social factors, and police-oriented factors.

In terms of social factors, scholars have confirmed a link between the accessibility of basic public services and a sense of safety. Some researchers have focused on the impact of pension insurance. They explore how pension insurance helps reduce poverty among older adults (11) and the mechanisms that influence aspects such as enhancing the happiness of the elderly (12). Studies on the mechanisms by which different types of basic public service equalization exert their influence have provided both theoretical foundations and empirical evidence for exploring the pathways through which these policies may affect residents' sense of safety.

While existing literature has acknowledged that police practices targeted at crime control and the suppression of illegal activities can shape residents' satisfaction with public security and further influence their perceived safety, relevant empirical investigations remain largely fragmented and context-specific. Empirical studies have particularly documented the significant role of visible police patrols in residential communities in mitigating residents' fear of crime. Nevertheless, prior research has paid insufficient attention to the causal pathways and intermediate mechanisms linking routine policing activities, public security satisfaction, and individual perceptions of safety. Furthermore, few studies have systematically distinguished between different dimensions of policing practices or examined their heterogeneous effects on residents' fear of crime under diverse social and environmental contexts. This study therefore seeks to address these gaps by explicitly unpacking the mechanism through which policing behaviors shape residents' sense of safety, with the aim of providing both theoretical refinement and empirically grounded evidence for understanding the social outcomes of policing (13). Therefore, residents' sense of safety is influenced by various factors at different levels and dimensions.

Based on the above literature review, most studies on basic public service equalization have focused on fairness, perceived gains, and happiness. Therefore, this study attempts to examine the specific influence of these policy reforms on residents' sense of safety. Based on previous research (14) and considering the convenience of obtaining data through the CSS questionnaire, a single-question approach was adopted to measure residents' sense of safety. Although there are subjective measurement errors, it can still conform to the actual perception of residents and has a more macroscopic measurement significance for the vague variable of security. Taking China as the research context, it explores how residents' sense of safety is influenced by different policy reforms across provinces. It also investigates the mechanisms and outcomes of such influence.

## 3 Theoretical framework and research hypotheses

**Figure 1:**
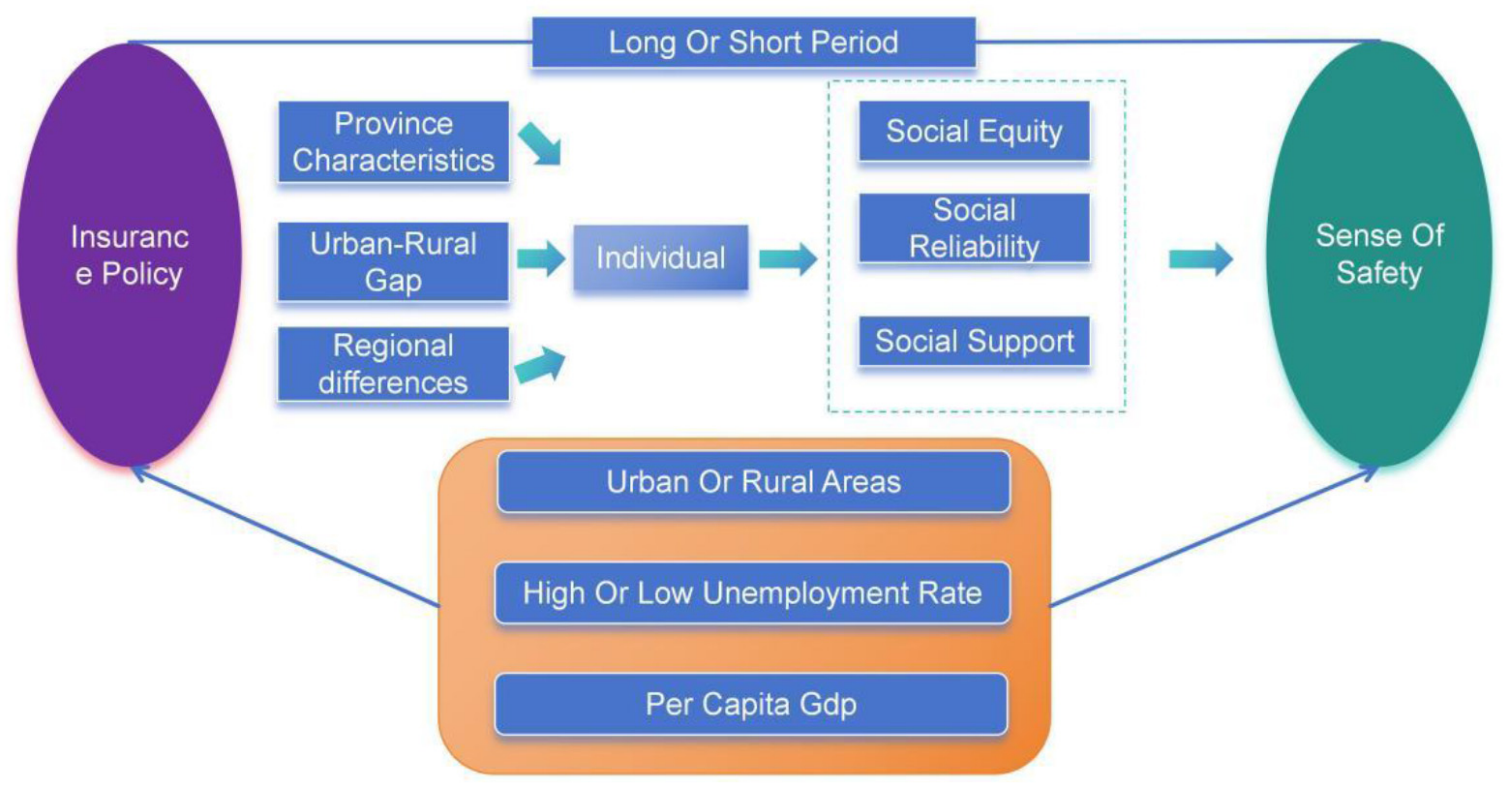
Mechanism of an insurance policy.

Medical insurance, unemployment insurance, and pension insurance are integral components of basic public service protection systems worldwide. Medical insurance influences residents' sense of safety by shaping their expectations regarding the treatment of physical health issues. Unemployment insurance provides financial compensation to residents facing job loss, which can potentially affect their sense of safety in daily life. Pension insurance represents an investment in future living security after retirement and, as part of social security system reforms, also affects residents' actual sense of safety in daily life. The specific mechanisms of influence are illustrated in Figure 1. Based on the existing literature (15), for the majority of citizens, particularly those in disadvantaged positions, the reduction of objective disparities fosters subjective well-being. When the government implements reforms to equalize access to essential services, it signals the fulfillment of the social contract and mitigates relative deprivation. This attenuation of perceived unfairness stabilizes future expectations and validates the legitimacy of the social order, thereby enhancing psychological safety (16). In the baseline regression, this study examines the impact of basic public service equalization—particularly social security policy reforms—on residents' sense of safety, and proposes two opposing hypotheses:

Hypothesis 1a: The reform of equalization of basic public services enhances residents' sense of safety by improving their perception of unfairness.
Hypothesis 1b: The reform of equalization of basic public services reduces residents' sense of safety by lowering their perception of unfairness.

The research framework of this article is as follows: it explores how individuals, under the influence of the national public service guarantee policies, are affected by factors such as provincial-level factors, urban-rural factors, various economic and social factors of the province where they reside, and individual economic and social behavioral choice factors, and through the enhancement of social fairness perception, social support perception, and social trust perception, they have formed the background for a positive improvement in residents‘ sense of safety in the long-term or short-term impacts. This has thus formed my research framework. Taking insurance participation in the basic public service equality as the manifestation of individuals' embedding in national behavior, a baseline regression is conducted on residents' sense of safety, and the economic and social heterogeneity factors are also tested. Using the concepts of social fairness, social trust, and social support, which are commonly associated with social security in existing sociological literature, as mediating variables, and considering the longitudinal influence of time, the impact of basic public service equality on residents' sense of safety is explored under the influence of spatial spillover effects in the long-term and short-term.

### 3.1 Social equity

Social security systems and policies can influence residents' sense of safety through multiple mechanisms, either directly or indirectly. First, by providing public services, they meet residents' basic living needs and reduce feelings of fear and threat in daily life. Some studies have shown that employment conditions and their potential impacts on perceptions of social injustice may exacerbate social conflicts. Such conflicts can, in turn, indirectly weaken people's sense of safety (17), underscoring how unemployment insurance can influence residents‘ sense of safety through its impact on perceptions of social equity. Other studies have shown that systemic inequalities in access to education, healthcare, and welfare can perpetuate a vicious cycle of insecurity and social deviance. Therefore, the social security policy promotes social fairness by improving basic public services and subsequently influences residents' safety perceptions, as supported by the literature.

However, the implementation of social security policy reforms may impose heavier daily living burdens on residents, thereby exacerbating their sense of insecurity. Variations in the timing of social security policy adjustments and modifications to policy content may diminish residents' perceptions of social equity, thereby elevating their perceived insecurity. Therefore, the influence of basic public service equalization on social equity and residents' sense of safety is inherently complex, which leads to the formulation of Hypothesis 2.

Hypothesis 2: The reform of equalization of basic public services enhances residents' sense of safety by alleviating their perception of unfairness.

### 3.2 Social trust

Social trust encompasses elements that influence interpersonal interactions, interpersonal trust, and government-citizen trust in modern societies. Trust between individuals occupying different social roles shapes residents' sense of safety through their mutual interactions. In addition, public trust in the government during crises can shape citizens' sense of safety. For instance, citizens' sense of safety can moderate the process by which online media influence public trust in the government amid crisis events [(18), pp. 10–12]. Then, basic public services may enhance residents' perceived social safety by strengthening connections between the public and the government. Trust in the government refers to citizens' expectations, evaluations, confidence, and satisfaction regarding the government's political processes, administrative conduct, and public service delivery. State institutions and government policies can shape people's perceptions of equity in their social communities or economic lives, and inequalities in market value distribution may give rise to a sense of relative deprivation, which may affect people's sense of happiness [(19), pp. 381–382]. Therefore, it can be concluded that there is a positive correlation between citizens' social trust and their sense of safety. This study thus proposes one hypothesis.

Hypothesis 3: The reform of equalization of basic public services promotes an increase in residents' sense of safety by improving their perception of unfairness.

### 3.3 Social support

Social support comprises three dimensions, namely tangible objective support such as material assistance, emotional subjective support including the understanding and respect perceived by individuals in social relationships, and the degree of individuals' utilization of social support (20). Existing research has demonstrated that social support can enhance individuals' self-efficacy while exerting a facilitative effect on their job satisfaction [(21), pp. 99–101]. Social support influences residents through social networks, including material and spiritual support. Therefore, the commonality of the social support dimension is the positive external resources that individuals obtain from the social environment, which can alleviate stress and enhance well-being. It bears a strong association with the dynamic interactions between individuals and society. At the same time, studies have shown that emotional support and psychological support included in social support reflect social tolerance (22). Research also shows that social support is related to an individual's perceived stress, and this societal influence on an individual's resilience affects personal well-being and life satisfaction (23). Therefore, this study considers social support as the effect of social resources on individuals' subjective perception, and thus uses the social inclusion dimension of the CSS questionnaire for measurement. The social support network can provide security for residents' life expectations and can reduce residents' risk perception within a certain period. Therefore, this study proposes one hypothesis:

Hypothesis 4: The reform of equalization of basic public services enhances social support for residents, which in turn leads to an improvement in residents' sense of safety.

### 3.4 Heterogeneous elements

Prior investigations reveal significant urban-rural developmental disparities in measuring the level and differentials of China's urban-rural integration. Specifically, gaps are observed between the integration of institutional construction and the on-the-ground implementation of institutional practices (24). Consequently, it is necessary to disaggregate analyses by urban and rural regions to assess the effectiveness of China's basic public service equalization policies. In China's regional development strategy, province regions are subject to distinct guiding orientations for development decisions within the strategic hierarchy. Given that the practices of social security policy reform vary across these regions, their respective impacts on residents' sense of safety also differ. For this reason, this study incorporates heterogeneous factors related to space and time in the analysis.

## 4 Research design

### 4.1 Model specification

#### 4.1.1 Baseline regression

This study employs the difference-in-differences (DID) method to estimate the effect of social security policy reforms for basic public service equalization on residents' sense of safety. This approach is chosen to accommodate the divergent policy rolling out schedules across pilot cities. The model is specified in detail below: Equation 1.


$$\begin{array}{l} {Safety}_{ijt}=\beta_{0}+\beta_{1}\left({Insurance}_{it}\times {Time}_{t}\right)+\lambda_{0}\sum_{i=1}^{n}{Control}_{it}\\ \;+\mu_{it}+\omega_{it}+\varepsilon_{it} \end{array}$$ (1)

In Equation 1, the subscripts i, j, and t denote province, individual, and time, respectively. *Insurance*_*it*_ is a dummy variable indicating the post-pilot period (1 = post-pilot period, 0 = pre-pilot period). The interaction term *Treat*_*it*_ × *Time*_*t*_ serves as the core explanatory variable capturing the policy effect. *Control*_*it*_ represents the set of control variables, μ_*j*_ and ω_*t*_ denote individual fixed effects and time fixed effects, respectively. ε_*ijt*_ is the stochastic error term. Standard errors are clustered at the provincial level security.

#### 4.1.2 Parallel trend test

The validity of the DID framework relies critically on the parallel trends assumption, which dictates that residents' sense of would evolve similarly in the treatment and control groups in the absence of the policy intervention. To formally test this identifying assumption through empirical analysis, we use the event-study framework. This procedure entails introducing interaction terms between the treatment group indicator and time-fixed effects for the periods preceding and following the social security reform into the benchmark model, as shown in the following specification (Equation 2).


$$\begin{array}{l} Y_{i}=\beta_{0}+\sum_{t=1+}^{4}y_{n}\times {pre}_{it}^{n}+\beta_{1}\times {current}_{it}\\ \;+\sum_{i=1}^{6}\delta_{n}{post}_{it}^{n}+\sum_{\gamma_{i}}{Control}_{it}\mu_{it}+\omega_{it}+\varepsilon_{it} \end{array}$$ (2)

*Pre*_*n*_ denotes the dummy variable for the years before policy implementation, *current*_*it*_ represents the implementation year, and *Post*_*n*_ denotes the dummy variable for the nth year after policy implementation. If the coefficients of the pre-policy terms *Pre*_*n*_ are statistically significant and positive, which may indicate estimation bias due to observed confounding factors. Conversely, the statistical insignificance of these coefficients bolsters the causal identification, indicating that all documented improvements in residents' sense of safety are attributable to the social security policy reforms.

### 4.2 Variable definitions and measurements

#### 4.2.1 Dependent variable

Residents' sense of safety is measured by their comprehensive evaluation of public conditions. Drawing on the calculation method from previous studies [(25), pp. 14–16], this study uses public safety evaluation as the measurement indicator for residents' sense of safety. Specifically, we adopt the question from the China Social Survey (CSS): “How would you rate the overall social safety situation in China at present?” The responses are coded on a 4-point scale: “very unsafe” = 1, “relatively unsafe” = 2, “relatively safe” = 3, and “very safe” = 4.

#### 4.2.2 Core explanatory variable

Our key independent variable is the binary indicator Insurance (INS), which captures the exposure of individual residents to the social security reform initiatives. To address potential endogenous biases, we operate this variable according to the policy rolling out schedule. By incorporating the connotations of basic public service equalization, it takes into account the three types of policies covering employment, medical care, and pension insurance [(26), pp. 24–26]. This study designates 2015 as the national policy implementation year and adjusts the policy implementation timing forward or backwards based on the concentrated years of policy reform in each province. Specifically, individuals living in cities that launched the targeted policy and enrolled in basic employee medical insurance, basic resident medical insurance, unemployment insurance, or pension insurance were designated as the treatment group (coded as 1). All remaining respondents constituted the control group (coded as 0). Both the control and treatment groups are derived from samples covering 31 provinces and municipalities directly under the Central Government in the China Social Survey (CSS).

#### 4.2.3 Control variables

Drawing on existing studies and considering the influencing factors of the level of basic public service equalization and residents' sense of safety [(27), pp. 4–7], the control variables include individual and provincial characteristics. Among these, Detailed definitions and summary statistics for the variables employed in this study are presented in Table 1.

**Table 1:** Measurement of key variable.

Variable type	Variable name	Definition
Dependent variable	Safety	The sense of safety is measured by using a five-level score based on the residents' assessment of the overall security situation of the society.
Core independent variable	Insurance	The timing of the government's social security system reform and the participation status of residents (1 for the treatment group, 0 for the control group)
Individual characteristics	Gender	Male = 1;Female = 0
	Age	Age of the respondents
	Education	The level of education is rated from 1 to 4. The higher the value, the higher the level of education.
	Marital Status	Married = 1; Unmarried = 0; Divorced and not remarried = 2; Divorced and remarried = 3; Widow and not remarried = 4; Widow and remarried = 6; Refused to answer = 8
Regional charateristics	Rural of Countryside	Rural = 1 Countryside =0
	Area	East area = 1 Middle area = 2 West area =3
Household characteristics	Family average expense	Average household consumption expenditure
Province characteristics	Percapita	Provincial per capita GDP figure
	Unemploymentrate	Provincial unemployment rate
	Industrial structure	Provincial industrial structure
	General fiscal revenue	Overall fiscal revenue of the province
	General fiscal expenditure	Overall fiscal expenditure of the province
	House prices	Average house price of the province
	Total disposable income	Average disposable income of urban and rural residents in the province
	Urban disposable income	Average disposable income of urban residents in provinces
	Countryside disposable income	Average disposable income of rural areas in the province
	Youth population ratio	Proportion of young people (aged 18-29) in the province
	Elderly population ratio	Proportion of elderly population in the province
Mechanism characteristics	Social trust	Using the ten-level measurement of social trust in the questionnaire, the higher the value, the greater the degree of trust.
	Social support	Using the ten-level measurement of social support degree in the questionnaire, the higher the value, the higher the degree of social support.
	Social equity	Using the ten-level measurement of social fairness in the questionnaire, the higher the value, the greater the degree of fairness.

### 4.3 Data description and descriptive

#### 4.3.1 Data description

Empirical data for this analysis are sourced from the China Social Survey (CSS). As a large-scale, national longitudinal sampling survey program, the CSS was initiated in 2005 by the Institute of Sociology of the Chinese Academy of Social Sciences. Currently publicly available, this dataset comprises 8 waves (2006, 2008, 2011, 2013, 2015, 2017, 2019, 2021), covering all 31 provincial-level administrative divisions in mainland China, including 151 districts, cities, and counties. Each survey interviewed 7,000 to 10,000 households, and the results are generalized to the household population aged 18 to 69 nationwide. To align with the actual status of residents‘ sense of safety and in conjunction with the implementation scope of China's basic public service equalization policies, the analytical sample underwent multi-step data processing: (1) Restricting the sample to individuals aged 18 to 69 years old, and removing observations with significant missing values in the dependent variable and core independent variables; (2) Excluding observations restricted by missing data on social support, social trust, and social equity in certain provinces; (3) Normalize the residents' sense of safety scores of the non-five-level measurements for the years 2006, 2008, 2011, and 2013, so that their values range between 1 and 4; (4) Defining the policy implementation time based on the year when large-scale social security reforms were rolled out in each province; (5) Minor missing values for independent variables are imputed through linear interpolation, with all continuous variables winsorized at the 1% level to reduce outlier bias. The final processed dataset is a panel dataset containing 72,247 valid observations across 8 periods. However, a significant imbalance existed between the control and treatment groups, with the latter accounting for 88.3% of the total sample. A 1:4 nearest-neighbor propensity score matching (PSM) strategy was adopted, and the results indicated no significant differences across most covariates, confirming successful sample balancing. The multicollinearity test yielded a mean variance inflation factor (VIF) of 3.22, suggesting the absence of significant multicollinearity among variables.

#### 4.3.2 Descriptive statistics

Table 2 reports the summary statistics for the key variables employed in this study. Among them, the core independent variable Insurance has a mean value of 0.775 and a variance of 0.417, indicating that residents on the whole tend to participate in insurance schemes. For the dependent variable, residents' sense of safety (measured on a 5-point scale) has a mean of 4.084, suggesting a relatively high level of perceived security, and a variance of 2.147, reflecting a high degree of variability across the sample. Regarding the mechanism variables, the mean values of social support, social trust, and social equity (measured on a 10-point scale) are 4.982, 4.892, and 4.0, respectively. These figures indicate that the three mechanisms in the sample are generally slightly below the midpoint of the scale, a result that may be associated with the varying conditions across different provinces and cities. In addition, indicators such as per capita GDP, industrial development level, total fiscal revenue, and total fiscal expenditure across provinces exhibit substantial variability, reflecting significant inter-provincial disparities. In contrast, individual and household characteristics exhibit relatively small fluctuations, indicating that the sample is well-balanced across these attributes.

**Table 2:** Descriptive statistics.

VarName	Mean	SD	Min	Median	Max
Insurance	0.775	0.417	0.000	1.000	1.000
Social trust	4.892	2.205	1.000	5.000	10.000
Social Support	4.982	2.472	1.000	5.000	10.000
Rural or countryside	0.624	0.596	0.000	1.000	5.000
Area	1.857	0.792	1.000	2.000	3.000
Social equity	4.000	2.320	1.000	3.000	10.000
Safety	4.084	2.147	1.000	3.000	5.000
Percapita	49,949.982	27,154.459	112.300	45,387.000	1.84e+05
Unemploymentrate	3.353	0.712	1.200	3.400	11.200

## 5 Empirical results

### 5.1 Baseline regression analysis

Starting with a quasi-natural experiment on the impact of basic public service equalization on residents‘ sense of safety, this paper constructs a difference-in-differences (DID) model to examine the effect of basic public service equalization on residents' sense of safety. Table 3 presents the baseline regression results for the influence of urban-rural basic public service equalization on residents' sense of safety in China. Column (1) displays the baseline regression results from specifications that exclude control variables. These estimates suggest that the rollout of basic public service equalization policies positively affects residents' sense of security, and this effect is statistically significant at the 1% threshold. We subsequently re-estimate the benchmark model by incorporating all control variables. As shown in Column (2), the coefficient of the DID interaction term retains its statistical significance at the 1% level with a value of 0.161 and maintains a positive sign. This empirical result is consistent with evidence from existing research.

**Table 3:** Results of baseline regress.

Variables	Sense of safety	
	(1)	(2)
DID	0.225^***^ (3.04)	0.161^***^ (2.97)
*marriage*		−0.105^***^ (−4.38)
Per capita		0.825^***^ (20.46)
Industrial structure		0.0154 (1.23)
General fiscal revenue		0.00248^***^ (23.71)
Fiscal expenditure		0.00100 (1.25)
Hospitals		0.000274 (0.92)
Physicians		0.0189 (0.92)
Hospital beds		0.0271 (0.24)
Total disposable income		−0.569^***^ (−9.22)
Urban disposable income		0.0000568^***^ (7.94)
Countryside disposable income		0.101^***^ (9.50)
Youth population ratio		0.777^*^ (1.88)
Elderly population ratio		0.766^***^ (3.63)
Year FE	Yes	Yes
Province FE	Yes	Yes
Year x Province FE	Yes	Yes
Constant	3.121^***^ (4.02)	2.495^***^ (5.19)
Observation	72,247	72,247
*R* ^2^	0.615	0.642

*T*-values in parentheses.

^*^p < 0.1,

^**^p < 0.05,

^***^p < 0.01. SEs clustered at city level. Year = time fixed effects, id = individual fixed effects (hereafter).

^**^represents that the p-value is less than 0.05, indicating that the significance holds within the 95% confidence interval. Meanwhile, the values in the brackets within the table represent the significance levels. Therefore, the asterisks in each set of numbers refer to correct data.

### 5.2 Endogeneity test

#### 5.2.1 Instrumental variable method

Due to various factors, such as a limited sample scope and omitted variables, the baseline results may be subject to endogeneity. To address this problem, this paper adopts the instrumental variable (IV) method. Based on previous research (28), this study selects the number of provincial hospitals as the instrumental variable. Since the number of provincial hospitals is determined by the economic capacity of the province's residents and constitutes government action, it manifests the provision of social security. It is not directly related to residents' sense of safety, and thus does not meet the theoretical criteria for selecting instrumental variables. in the province for the year as a mediating variable, where the number of hospital constructions in the province is derived from the provincial statistical yearbook of that year. As shown in Table 4, the first-stage regression results [see Column (1) of Table 4] reveal that the regression coefficient of the number of hospital constructions per province in the IV regression is 0.067, which is statistically significant at the 1% level, and consistent with the coefficient direction in the baseline regression. For the weak instrumental variable test, the Kleibergen-Paap rk LM statistic equals 72.49, and the Anderson-Rubin (AR) test yields a significant result (*P* = 0.000 < 0.1), indicating the absence of weak instrumental variable problems. The Cragg–Donald Wald *F* statistic is 39.35, which exceeds the Stock–Yogo critical value of 16.38 at the 10% significance level, confirming the exogeneity of the instrumental variable. Collective results from the preceding tests confirm the appropriateness of our selected instrumental variable. The second-stage regression outcomes are documented in Column (2) of Table 4. The coefficient for the Insurance variable is 0.0064 and is statistically significant at the 1% level. This evidence demonstrates that basic public service equalization policies improve residents' sense of safety, empirically validating Hypothesis 1.

**Table 4:** Endogeneity test results.

Variables	(1)	(2)	(3)	(4)	(5)
	Stage1	Stage2	Mahalanobis	Radius	Kernel
iv	0.067^***^ (9.47)				
Insurance		0.0061^***^ (3.77)	0.064^***^ (3.37)	0.061^***^ (4.19)	0.063^***^ (3.43)
Cragg–Donald Wald F statistic	39.35				
Kleibergen–Paaprk LM statistic	72.49^***^				
Control variables	Yes	Yes	Yes	Yes	Yes
Year FE	Yes	Yes	Yes	Yes	Yes
Province FE	Yes	Yes	Yes	Yes	Yes
Year × City FE	Yes	Yes	Yes	Yes	Yes
Observation	72,247	72,247	72,247	72,247	72,247
*R* ^2^	0.761	0.420	0.237	0.551	0.436

*T*-values in parentheses.

^*^p < 0.1,

^**^p < 0.05,

^***^p < 0.01. SEs clustered at city level. Year = time fixed effects, id = individual fixed effects (hereafter).

^***^represents that the p-value is less than 0.01, indicating that the significance holds within the 99% confidence interval. Meanwhile, the values in the brackets within the table represent the significance levels. Therefore, the asterisks in each set of numbers refer to correct data.

#### 5.2.2 PSM-DID

Regional heterogeneity in the foundations of basic public service equalization policies may lead to selection bias in baseline regression results. To tackle this concern, this study employs the PSM-DID method to verify the robustness of the baseline regression results. The control variables from the baseline regression are used as covariates, and Mahalanobis distance, radius, and kernel matching are applied for sample matching. Control groups are identified for cities that have implemented basic public service equalization reforms, and the control groups obtained through this approach are closer to random selection. Columns (3) to (5) of Table 4 report the regression results. Specifically, the estimated coefficients for insurance derived from the three matching methods are 0.062, 0.061, and 0.063, respectively. These coefficients are consistent in direction with those from the baseline regression and are all statistically significant at the 1% level, confirming that basic public service equalization policies contribute to improvements in residents' sense of safety.

### 5.3 Robustness tests

#### 5.3.1 Parallel trend sensitivity test

**Figure 2:**
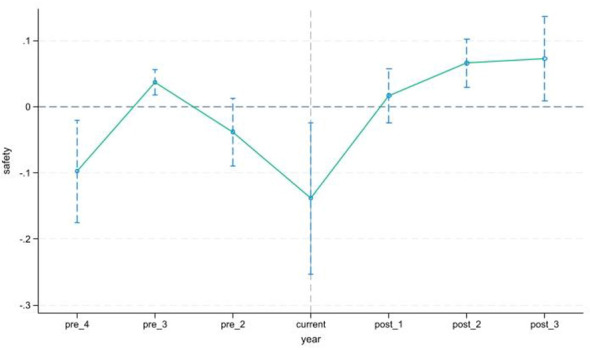
Parallel trend test results of residents' sense of safety.

Guided by the parallel trends assumption, this study employs an event study methodology to examine the impact of basic public service equalization policies on residents' safety. We adopt the period immediately preceding the policy rollout as the reference group to avoid multicollinearity, maintaining consistency with the baseline regression specifications. With 2015 as the benchmark year, this study applies the event study approach to analyze residents' sense of safety levels across the four pre-2015 periods, namely 2006, 2008, 2011 and 2013, and the three post-2015 periods, namely 2017, 2019 and 2021. The results of the pre-treatment trend analysis are presented in Figure 2. Specifically, when *t* < 0, the estimated coefficients β_*t* fluctuate slightly but remain mostly negative, suggesting no pronounced differences between the treatment and control groups in the pre-policy period of basic public service equalization. After the policy implementation in 2015, the policy effect on residents' sense of safety initially showed a downward trend, yet such an effect did not exhibit a rapid accumulation pattern. The average treatment effects rebounded in the second and third years following policy implementation, thus verifying that the parallel trends assumption holds.

#### 5.3.2 Exclude other competing hypotheses for testing

Previous sections have verified the influence of basic public service equalization on residents' sense of safety. However, multiple policies were successively issued by the central, provincial and municipal governments during the sample observation period. It is therefore necessary to further verify whether other competing policies may interfere with the regression results. In view of this, this study selects 10 provinces and municipalities that launched the expansion of work-related injury insurance coverage starting from 2011 and 6 provinces that implemented the Long-Term Care Insurance system since 2013, as well as the four provinces where the 2013 New Rural Cooperative Medical Insurance policy was extensively implemented, and the provinces that implemented the two basic education guarantee policies in 2015. These samples are incorporated into the baseline regression model based on their respective implementation years. As shown in Column 1 of Table 5, after excluding the interference from the aforementioned policies, the regression coefficient is 0.036 and statistically significant at the 5% level. This finding is broadly consistent with the baseline regression results, providing additional validation that basic public service equalization policies foster improvements in residents' sense of safety.

**Table 5:** Robustness test results.

	(1)	(2)	(3)	(4)
	Exclude other competing hypotheses for testing	Change the calculation method	Expected effect	Dual machine learning
**Insurance**	0.036^**^ (2.57)	0.061^***^ (4.22)	0.076^**^ (2.01)	0.047^***^ (3.16)
**RSS**	−0.162 (−0.358)			
**LTI**	−0.072 (−0.63)			
**Pre1**			−0.601 (−0.148)	
**Pre2**			−0.592 (−0.147)	
**Year FE**	Yes	Yes	Yes	Yes
**Province FE**	Yes	Yes	Yes	Yes
**Year** **×City FE**	Yes	Yes	Yes	Yes
**Control variables**	Yes	Yes	Yes	Yes
**Observations**	72,247	72,247	72,247	72,247
***R** ^**2**^*	0.367	0.024	0.439	

*T*-values in parentheses.

^*^p < 0.1,

^**^p < 0.05,

^***^p < 0.01. SEs clustered at city level. Year = time fixed effects, id = individual fixed effects (hereafter).

^*^represents that the p-value is less than 0.1, indicating that the significance holds within the 90% confidence interval. Meanwhile, the values in the brackets within the table represent the significance levels. Therefore, the asterisks in each set of numbers refer to correct data.

#### 5.3.3 Re-measurement of residents' sense of safety

This article calculates residents' sense of safety by averaging the seven dimensions (property safety, personal safety, transportation, medical care, food, labor, and privacy security) from each year's questionnaires using CSS. The formula is as follows.


$$\begin{array}{c} {Safety}_{new}=\frac{1}{n_{k}}\sum_{i=1}^{n_{k}}X_{ki} \end{array}$$ (3)

Here, *n*_*k*_ denotes the number of items in the sub-dimensions, and *X*_*ki*_*i* represents the score given by respondents to the items in the kth dimension. The regression results are presented in Column 2 of Table 5. The regression coefficient for insurance is 0.061, and its direction and statistical significance are consistent with those of the baseline regression. This indicates that the impact of basic public service equalization policies on residents' sense of safety remains consistent across measurement methods and remains statistically significant, thus verifying the reliability of the conclusion.

#### 5.3.4 Expected effect

Preliminary preparations, including pension insurance consolidation and the optimisation of unemployment insurance benefits, had been carried out prior to the implementation of the basic public service equalization policy. Therefore, this study advances the pilot implementation by 1 and 2 years and constructs the corresponding policy variables. The baseline model incorporates the one-year pre-pilot dummy variable *pre* 1 and the two-year pre-pilot dummy variable *pre* 2 to exclude control for anticipation effects that may confound the impact of the dual environmental policy constraints. The results are presented in Column 3 of Table 5. It can be observed that the regression coefficient of *Insurance* is 0.076 and statistically significant at the 5% level, while the regression coefficients of the dummy variables *pre* 1 and *pre* 2 are both statistically insignificant. It is indicated that the policy constraints' impact is not subject to interference from anticipation effects.

#### 5.3.5 Dual machine learning

Dual machine learning overcomes the limitations of traditional causal inference and standard machine learning by using regularization for variable selection, orthogonalization to reduce bias, and cross-validation to prevent overfitting. This framework effectively handles non-linear relationships among economic variables while constructing robust confidence intervals. This study uses a 1:6 split ratio to divide the dataset into the training set and the prediction set, and re-estimates the inhibitory effect of dual environmental policy constraints. The results are presented in Column 4 of Table 5. The data show that the estimated coefficient of *Insurance* after model adjustment is 0.047 and statistically significant at the 1% level, with the coefficient direction consistent with that of the baseline regression discussed earlier, which verifies the robustness of the estimates of this study.

### 5.4 Placebo test

**Figure 3:**
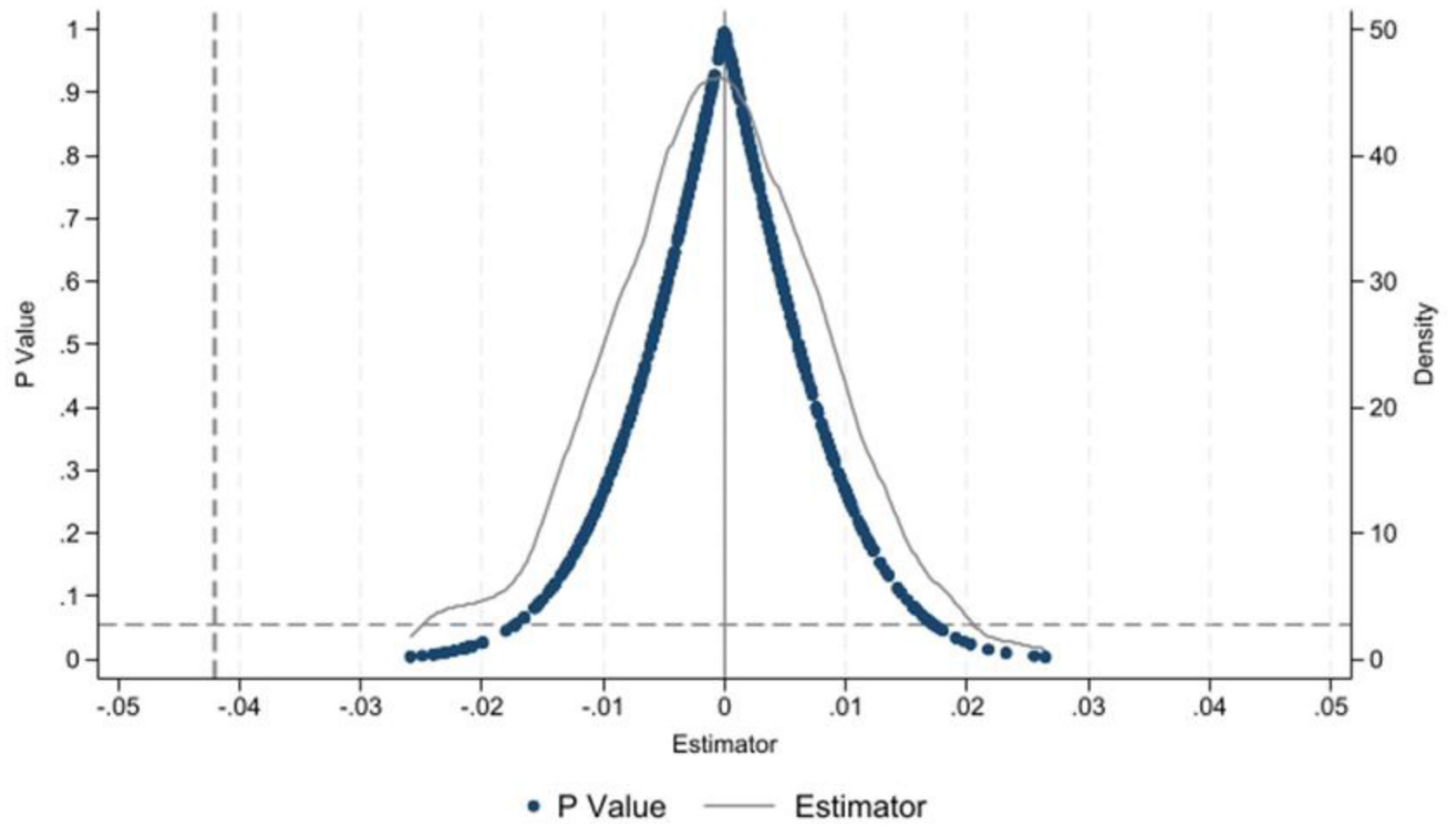
Placebo test results.

To further address and eliminate concerns that the study results might be driven by the policy rather than pre-existing time trends, we conduct a time-and-space-mixed simulation placebo test. This test is designed to evaluate whether significant disparities exist between the treatment and control groups prior to policy implementation and also addresses the potential issue of omitted-variable bias. The detailed implementation steps are outlined below in Figure 3. A number of individuals are randomly sampled without replacement from the analytical sample to act as pseudo-treated observations, and a common pseudo-policy intervention time is randomly designated. A subsequent difference-in-differences estimation is performed to generate an estimate of the placebo effect. The estimated effect of basic public service equalization on residents' sense of safety in this study is 0.161, which falls in the right tail of the placebo distribution and is an extreme outlier. It confirms the effectiveness of basic public service equalization in promoting residents' sense of safety.

## 6 Further analysis

### 6.1 Examination of mechanisms

To further explore the channels through which basic public service equalization exerts a positive influence on residents‘ sense of safety and verify whether the empirical results are consistent with the former proposes proposed in this study, we identify three mediating pathways—social trust, social equity, and social participation—through which basic public service equalization policies affect residents' sense of safety, based on previous research [(29), pp. 15–20]. These terms are used to explore the internal mechanism underlying the impact of relevant policies on residents' sense of safety by examining each link in the causal chain from basic public service equalization to residents' sense of safety. Drawing on the approach of the relevant study [(30), pp. 75–77]. The verification model is presented in equation 4. In the equation, **Med**_**it**_ denotes the mechanism variable, reflecting the mechanistic effects of basic public service equalization policies exerted through various channels. By separately calculating the equalization of basic public services and the mediating variables, the mediating effect was verified.


$$\begin{array}{l} Med_{it}=\beta_{0}+\beta_{1}Insurance_{it}+\lambda_{0}\sum_{i=1}^{n}Control_{it}+\mu_{it}\\ \;+\omega_{it}+\varepsilon_{it} \end{array}$$ (4)

$$\begin{array}{l} Safety_{it}=\beta_{0}+\beta_{1}Insurance_{it}+\beta_{2}Med_{it}+\lambda_{0}\overset{n}{\underset{i=1}{\sum }}Control_{it}+\mu_{it}\\ \;+\omega_{it}+\varepsilon_{it} \end{array}$$ (5)

#### 6.1.1 Social trust

In this study, the social trust variable is constructed as the mean score of residents' trust in the central government (CG), county and district governments (COG), and township and town governments (COTG), based on data from the China Social Survey (CSS). This study first confirms that residents' sense of safety is influenced by social trust. Moreover, social trust is shaped by social networks, and basic public service equalization itself constitutes a component of social relations. By strengthening connections between the government and citizens and enhancing mutual social trust among citizens through participation in public affairs facilitated by relevant policies, basic public service equalization exerts its impact on residents' sense of safety through the channel of social trust.

(1) represents the regression results of Model (4), showing that the coefficient of social trust is 0.210, which is significant at the 5% level, indicating that the implementation of equalization of basic public services has enhanced residents' perception of social trust. (2) represents the regression results of Model (5), where an intermediary variable is added in the baseline regression for social trust. In social trust, the coefficient of the independent variable insurance is 0.132, and the result is significant at the 1% level, being lower than the baseline regression coefficient of 0.225. The coefficient for Safety is 0.189, indicating that social trust plays a role in the process of basic public service guarantees influencing residents' sense of safety—these findings indicate that basic public service equalization policies enhance residents' positive sense of safety by promoting social trust. This might be because the equalized services have reduced “relative poverty” and “the sense of exclusion” caused by the uneven distribution of resources. According to the equity theory, when individuals perceive that the input-output ratio between themselves and the reference group is reasonable, their sense of unfairness decreases.

#### 6.1.2 Social equity

According to previous research [(31), pp. 38–40], perceptions of social equity affect residents' sense of safety. Social equity consists of equal opportunity and equitable outcomes. Drawing on previous studies (32), public welfare provided by the government and social factors are closely associated with social equity. As outcome-oriented factors, these elements represent the equity of outcomes delivered through proactive government actions. Therefore, this study argues that social equity perception also serves as a channel through which basic public service policies influence residents' sense of safety. It is measured using responses to the question about residents' evaluation of overall social equity and justice in the CSS questionnaire.

The results show that the coefficient of social equity in column (1) is 0.133, which is significant at the 1% level, indicating that the implementation of equalization of basic public services has enhanced residents' perception of social equity. In column (2), the coefficient of social equity for Safety is 0.113, suggesting that social equity perception plays a role in the impact of basic public service guarantees on residents' sense of safety, indicating that basic public service equalization policies enhance residents' sense of safety by improving their perceptions of social equity. This might be that when public services are provided fairly, not only do the material conditions improve, but more importantly, social relationships are also restructured. The establishment of government trust reduces the fear of the future because individuals believe that there is a system in place to provide support when facing risks. A fair environment reduces the antagonistic sentiments among social groups. When people are at similar levels of protection, their suspicion toward each other decreases, thus making them feel safer in social interactions.

#### 6.1.3 Social support

Drawing on previous research [(33), pp. 35] on the relationship between social support and sense of safety, it can be concluded that social support affects social security. Meanwhile, social support itself is an outcome of basic public service equalization policies, fostering both tangible and emotional social support. Therefore, this study incorporates social trust, social equity, and social support as mechanism variables to explore the underlying influence channels. Social support is measured using responses to the question about residents' perceived level of social tolerance in the CSS questionnaire.

The results in Table 6 show that the coefficient of social support in column (1) is 0.395, which is significant at the 1% level, indicating that the implementation of equalization of basic public services has enhanced residents' perception of social support. In column (2), the coefficient of social support for Safety is 0.291, which is lower than the reduction, suggesting that the perception of social support plays a role in the impact of basic public service guarantees on residents' sense of safety. This might be because the equalization of public services, by improving the community environment and public facilities, has facilitated community interaction, thereby strengthening the social support network. Equalized public services (such as community elderly care and public safety facilities) are themselves a form of formal social support. At the same time, they promote harmonious neighborhood relations and enhance informal social support. A strong social support network can provide practical resources, making individuals feel “supported”, thereby objectively reducing vulnerability and enhancing security.

**Table 6:** Examination of mechanisms.

Variables	(1)	(2)	(3)	(4)
	Insurance	Sense of safety (social trust)	Sense of safety (social equity)	Sense of safety (social support)
Insurance	^−^	0.132^***^ (3.11)	0.145^**^ (2.89)	0.142^***^ (3.75)
Social trust	0.210^**^ (2.05)	0.189^**^ (2.97)	^−^	^−^
Social equity	0.133^***^ (5.14)	^−^	0.113^***^ (7.45)	^_^
Social Support	0.395^***^ (4.53)	^_^	^_^	0.291^***^ (3.15)
Constants	2.590^***^ (4.17)	1.248^***^ (4.05)	1.743^***^ (5.01)	1.948^***^ (3.97)
Year FE	Yes	Yes	Yes	Yes
Province FE	Yes	Yes	Yes	Yes
Year × Province FE	Yes	Yes	Yes	Yes
Control variables	Yes	Yes	Yes	Yes
Observations	72,247	70,025	70,025	70,025
*R* ^2^	0.642	0.785	0.791	0.773

*T*-values in parentheses.

^*^p < 0.1,

^**^p < 0.05,

^***^p < 0.01. SEs clustered at city level. Year = time fixed effects, id = individual fixed effects (hereafter).

^*^represents that the p-value is less than 0.1, indicating that the significance holds within the 90% confidence interval. Meanwhile, the values in the brackets within the table represent the significance levels. Therefore, the asterisks in each set of numbers refer to correct data.

In summary, basic public service equalization policies enhance residents' sense of safety by improving their perceptions of social trust, social equity, and social support. These three mechanisms each play a partial mediating role in this process, which is consistent with the expected results of the hypotheses.

### 6.2 Heterogeneity analysis

#### 6.2.1 Urban and rural areas

To further analyze the areas of urban and rural basic public service equalization policy, This paper incorporates the divergent developmental disparities in basic public service equalization between urban and rural areas in China, as well as the impact of the urban-rural dual system, disparities in living standards, and varying degrees of social security system implementation on the mechanism of basic public service equalization policies, as noted in previous research [(34), pp. 100–101]. Drawing on the two distinct development contexts of urban and rural China, this study explores the influence of basic public service equalization policies on residents' sense of safety in urban and rural areas. Grouped regression analyses are conducted separately for the urban and rural resident samples.

Columns 1 and 2 of Table 7 demonstrate that although the insurance coefficients are significantly positive across both groups, the policy's enhancement effect is substantially more pronounced in urban areas. It may be attributed to the more thorough implementation of basic public service policies in urban regions.

**Table 7:** Heterogeneity analysis results.

Variables	(1)	(2)	(3)	(4)	(5)	(6)
	Urban area	Countryside area	High unemployment rate	Low unemployment rate	High per capita GDP	Low per capita GDP
Insurance	0.125^***^ (3.04)	0.092^***^ (2.93)	0.138^***^ (4.03)	0.091^***^ (5.04)	0.107^***^ (3.22)	0.128^**^ (2.24)
Constants	2.478^***^ (3.18)	2.233^***^ (4.21)	2.53^**^ (2.20)	2.433^**^ (1.97)	2.605^***^ (3.16)	2.155^**^ (2.28)
Controls	Yes	Yes	Yes	Yes	Yes	Yes
Year FE	Yes	Yes	Yes	Yes	Yes	Yes
Province FE	Yes	Yes	Yes	Yes	Yes	Yes
Year × Province FE	Yes	Yes	Yes	Yes	Yes	Yes
Observations	39,460	28,806	39,122	33,125	31,677	40,570
*R* ^2^	0.651	0.633	0.605	0.650	0.664	0.590

*T*-values in parentheses.

^*^p < 0.1,

^**^p < 0.05,

^***^p < 0.01. SEs clustered at city level. Year = time fixed effects, id = individual fixed effects (hereafter).

^*^represents that the p-value is less than 0.1, indicating that the significance holds within the 90% confidence interval. Meanwhile, the values in the brackets within the table represent the significance levels. Therefore, the asterisks in each set of numbers refer to correct data.

The deep-rooted cause of this difference lies in the development imbalances and varying conditions for policy implementation under the long-standing urban-rural dual structure. Based on previous studies (35), from the perspective of policy implementation foundations, urban areas, with their stronger fiscal capacity, well-established public service infrastructure, and efficient grassroots governance systems, provide solid support for policy implementation. Policies aimed at equalizing basic public services can be more effectively promoted, with broader service coverage and more effective implementation, enabling policy benefits to reach residents quickly and noticeably enhance their sense of safety. In contrast, rural areas are constrained by factors such as dispersed population distribution, insufficient fiscal investment, and relatively weak grassroots governance capabilities. Even when equalization policies are advanced, some remote areas still face problems such as incomplete service coverage, lower implementation efficiency, and uneven service quality, which, to some extent, diminish the efficacy of these policies in improving the sense of safety. From the perspective of residents' perceptions, urban residents have more accessible channels for obtaining public services and greater policy awareness, making them more sensitive to service improvements driven by policies. Meanwhile, some rural residents are affected by information asymmetry, leading to insufficient understanding of policies and difficulty in fully perceiving policy benefits, further widening the gap in policy effects between urban and rural areas.

#### 6.2.2 Unemployment rate

Drawing on the research before [(36), pp. 15], it can be observed that the unemployment rate is correlated with the formulation of social security policies and influences the design of basic public service policies during a given period. Therefore, the unemployment rate is a factor that needs to be considered when exploring the influence of basic public service equalization on residents' sense of safety. This study divides the sample into two groups: high-unemployment and low-unemployment areas.

The results presented in Columns 3 and 4 of Table 7 demonstrate that social security reform policies have a greater impact on provinces and municipalities directly under the Central Government with high unemployment rates. This indicates that basic public service equalization exerts a more pronounced positive impact on the sense of safety of residents in areas with high unemployment rates, which may be attributed to the role of unemployment insurance in enhancing residents' perception of economic security.

The core reason for this difference lies in residents' varying risk perceptions and demand orientations across different unemployment rate environments, leading to divergent policy adaptability. Residents in high-unemployment areas face greater employment uncertainty and income fluctuation risks, leading to a more urgent need for public services such as basic living security and employment support, making them a typical “security-oriented demand” group. Based on previous studies (37), Policies aimed at equalizing basic public services—through measures such as improving unemployment insurance benefits, expanding the coverage of employment skills training, and strengthening the linkage between social security and employment assistance—can directly alleviate this group's economic anxiety and survival pressures, solidify the bottom line of life security, and consequently significantly enhance their sense of economic safety and overall safety. In contrast, residents in low-unemployment areas have stable sources of income and face lower employment risks, and their demand for public services has shifted from “basic security” to “quality improvement.” Equalization policies, which focus on balancing basic services, are less well aligned with this group's needs, naturally resulting in weaker marginal policy effects. Moreover, high-unemployment areas are often accompanied by a certain level of social anxiety. By equitably allocating public resources, equalization policies can also enhance a sense of social fairness and government credibility, thereby indirectly amplifying the positive influence on safety.

#### 6.2.3 Per capita

The per capita GDP of provinces/municipalities directly under the Central Government itself reflects the level of economic development and population carrying capacity of the corresponding regions, and it affects the coverage and support intensity of basic public service equalization policies. This study sets the average per capita GDP of all sampled provinces/municipalities as the threshold; those above the average are classified as provinces/municipalities with high per capita GDP, and those below are classified as provinces/municipalities with low per capita GDP.

The results are presented in Columns 5 and 6 of Table 7. It can be observed that basic public service improving policies exert a far more pronounced promoting effect on provinces with low per capita GDP. It may be attributed to the higher marginal improvement effect of such policies on residents' sense of safety in regions with relatively underdeveloped economic capacity.

The essence of this difference lies in the varying degrees of alignment between regional economic development stages and policy supply orientations, leading to differentiated marginal effects of public services. Regions with low per capita GDP are mainly in the economic catch-up stage, have limited fiscal capacity, and have long suffered from insufficient supply of basic public services, such as healthcare, education, and employment. These areas are characterized by a shortage of “survival-oriented” public services, and residents have extremely urgent needs for basic protection. The core of public service equalization policies is to address shortcomings in fundamental services, which precisely match the core needs of residents in low per capita GDP areas. The improvement in services brought about by these policies can quickly fill the demand gap, with a more substantial marginal effect on residents' sense of safety. In contrast, regions with high per capita GDP have a relatively well-developed basic public service system, and residents' demand for public services has shifted from “survival-oriented” to “development-oriented,” focusing more on personalized, high-quality, and diversified services, such as premium educational resources and high-end medical services. Equalization policies, which focus on narrowing gaps in basic services, are less able to meet the high-level demands of residents in these areas, so their impact on enhancing residents' sense of safety is relatively limited. Additionally, residents' sense of safety in high-GDP regions is more influenced by factors such as career development, property security, and quality of life, further reducing the marginal contribution of basic public service equalization policies.

### 6.3 Analysis of spatial overflow effect

From the perspective of the influence of basic public service equalization supply, such policies not only exert an influence on residents' sense of safety within the region but may also indirectly affect neighboring areas. Among them, the global Moran index was used to test the spatial autocorrelation of agricultural carbon emission efficiency. The relevant formula is as follow.


$$\begin{array}{l} Safety_{it}=\rho WSafety_{it}+\delta_{0}+\delta_{1}Insurance_{it}+WInsurance_{it}\theta \\ \;+\delta_{2}Control_{it}+\mu_{it}+\omega_{it}+\varepsilon_{it} \end{array}$$

ρ*WSafety*_*it*_ represents the spatial lag term of the dependent variable, measuring the influence of the dependent variable values in neighboring regions on the dependent variable in the current region. δ represents the coefficient of the neighboring area's impact on the local area. *WInsurance*_*it*_θ represents the spatial lag term of the independent variables. This is the most distinctive part of the SDM model. The coefficient θ measures the influence of the independent variables in neighboring regions on the dependent variable in the current region. Here, W is the spatial weight matrix, quantifying the “proximity” relationship between different spatial units. When θ = 0, the SDM degenerates into the spatial lag model (SLM). When θ + ρδ=0, the SDM degenerates into the spatial error model (SEM).

The baseline regression results in Table 8 indicate that basic public service equalization can improve residents' sense of safety in the local region, as the significance *p*-values of the global Moran index from 2006 to 2021 were 0.000, 0.001, 0.001, 0.000, 0.001, 0.000, 0.002, and 0.002 respectively. All of them indicate significant results. In reality, however, neighboring regions maintain close economic ties, and social security policies in some provinces and cities have gradually become interconnected. The results of the model selection test for the spatial spillover effect are as follows in Table 9. Both the LM test and the Robust-LM test were significant, indicating the presence of spatial error effects and spatial lag effects. Thus, the spatial Durbin model (SDM) was initially selected. In the joint significance test, both the individual fixed effect and the time fixed effect were significant at the 1% level. Therefore, a two-way fixed effect model was adopted. Both the Wald test and the LR test were significantly able to reject the null hypothesis that the SDM degenerated into a spatial error model (SEM) or a spatial lag model (SAR). This confirmed that the SDM was the optimal choice.

**Table 8:** 2006 global moran index of residents' sense of safety in China.

Years	*I*	*Z*	*p*-value
2006	0.1262	4.8387	0.000
2008	0.1703	5.7634	0.001
2011	0.1715	6.0035	0.001
2013	0.1458	4.6388	0.000
2015	0.1182	4.4321	0.001
2017	0.0992	3.9054	0.000
2019	0.0853	3.6492	0.002
2021	0.0753	3.1732	0.002

**Table 9:** Ordinary panel models lm, lr, wald, and joint significance test.

Test	Statistical quantity
LM(error)	4.198^**^
Robust LM(error)	7.032^***^
LM(lag)	4.901^**^
Robust LM(lag)	7.863^***^
LR(sdmsar)	28.78^***^
Wald(sdmsar)	29.13^***^
LR(sdmsem)	17.96^**^
Wald(sdmsem)	21.84^***^
Joint significance (individual)	18.64^**^
Joint significance (time)	604.54^***^

^*^p < 0.1,

^**^p < 0.05,

^***^p < 0.01.

Table 10 presents the effect decomposition results of the SDM model. The direct effect of equalization of basic public services is 0.289, and the indirect effect is 0.804. Both have passed the significance tests at 10% and 5% levels, indicating that whether in the local area or neighboring areas, the application of equalization of basic public services will enhance residents' sense of safety. This may be due to the fact that equalization of basic public services is inherently part of the people's livelihood needs, and at the same time, the people's livelihood policies of one province will interact with those of other provinces. For example, medical insurance mutual recognition, and local payment for old-age insurance, etc. The total effect of the Insurance variable is 1.190, which has passed the significance test at 1% level, and is consistent with the direction of the direct effect and the indirect effect. Moreover, the effect is more significant. The total effect reflects the degree of equalization of basic public services in a specific province and the average impact level on the sense of safety of residents in the region. The results show the positive impact of equalization of basic public services in a province on the sense of safety of residents. Therefore, promoting equalization of basic public services has an enhancing effect on the sense of safety of residents both within the province and between provinces.

**Table 10:** Analysis of the time-space effect.

Variables	(1)	(2)	(3)	(4)	(5)
	Coefficient	Spatial lag term	Direct effect	Indirect effect	Overall effect
Insurance	0.362^**^ (0.141)	1.223^***^ (0.340)	0.289^*^ (0.134)	0.804^**^ (0.267)	1.190^***^ (0.263)
Control Variables	0.421^**^ (0.139)	0.008 (0.358)	0.424^**^ (0.135)	0.133 (0.269)	0.289 (0.263)
Observations	72,247	72,247	72,247	72,247	72,247
Year FE	Yes	Yes	Yes	Yes	Yes
province FE	Yes	Yes	Yes	Yes	Yes
Year × province FE	Yes	Yes	Yes	Yes	Yes
*R* ^2^	0.157	0.157	0.157	0.157	0.157

The values in parentheses represent the standard error; rho = −0.488, sigma2 = 0.006.

^*^p < 0.1,

^**^p < 0.05,

^***^p < 0.01.

### 6.4 Long-term and short-term effects

Long-term and short-term effects are important temporal dimensions that reflect the impacts of basic public service policies. To verify the short- and long-term effects of basic public service equalization policies, Drawing on the approaches of relevant studies (38) Considering the characteristics of regional correlation and policy supply interaction, this study adopts the triple difference-in-differences (SDID) method to address the deficiency of multi-period difference-in-differences in accounting for the linkage effect of policy supply. Based on the triple difference model, the interaction terms between Insurance and Long and Insurance and Short were constructed as Insurance × Long and Insurance × Short. This study defines the first and second years following the implementation of the reform in pilot provinces as the short-term period, with the dummy variable Short coded as 1; otherwise, it is 0. The third year and beyond constitute the long-term period, where the dummy variable Long takes a value of 1; otherwise, it takes 0.

Based on the triple difference-in-differences model, this study constructs interaction terms between the core variable and the time dummy variables, namely Insurance^*^short and *Insurance*×*Long*.

The results are presented in Columns 5 and 6 of Table 11. The data show that the coefficients for the two interaction terms are 0.599 and 1.863, both statistically significant at the 1% and 5% levels, respectively. These findings indicate that the policy tends to exert a relatively favorable effect in the short term. In the long term, however, the policy effect may be slightly weakened due to the attenuation of policy impacts and the emergence of new issues.

**Table 11:** Analysis of the long-term and short-term effect.

Variables	(1)	(2)
	Long	Short
Insurance × Long	0.599^**^ (1.99)	
Insurance × Short		1.863^***^ (2.93)
Control Variables	3.121^***^ (0.023)	3.121^***^ (0.023)
Observations	72,247	72,247
Year FE	Yes	Yes
province FE	Yes	Yes
Year × province FE	Yes	Yes
*R* ^2^	0.615	0.615

^*^p < 0.1,

^**^p < 0.05,

^***^p < 0.01.

## 7 Conclusion and policy implications

### 7.1 Conclusions

Based on the nationwide urban-rural fundamental public service equalization policy reform launched in China in 2015, this paper explores its impact on residents' sense of safety. Treating these two variables as a quasi-natural experiment, this study conducts an in-depth analysis of the effect and mechanism of urban and rural basic public service affecting residents' sense of safety, using data from the 2006–2021 China General Social Survey (CGSS) covering 34 provinces, autonomous regions, and municipalities directly under the Central Government. On the basis of the above research findings, conclusions are drawn:

(1) On the whole, the implementation of basic public service equalization policies exerts a significant positive promoting effect on residents' sense of safety across all provinces and cities in China. Hypothesis 1a is thus verified, while Hypothesis 1b is rejected.
(2) Mechanism analysis shows that basic public service equalization policies affect residents' sense of safety by enhancing their social trust in the government and interpersonal relationships, which verifies Hypothesis 2. Such policies also exert a positive influence on residents' sense of safety through the channel of social support, confirming Hypothesis 3; and they positively influence residents' sense of safety by improving their perception of social equity, thus validating Hypothesis 4.
(3) Heterogeneity analysis indicates that basic public service equalization policies not only exert a positive impact on residents' sense of safety, but also show heterogeneous effects across different contexts. In terms of urban and rural regions, rural areas are more susceptible to the impacts of equalization policy reform than urban areas. In terms of unemployment rates, regions with higher unemployment rates are more likely to see an improvement in residents' sense of safety under the influence of basic public service equalization policies than low-unemployment regions. Meanwhile, regions with low per capita GDP are more responsive to such policies in boosting residents' sense of safety than regions with high per capita GDP.
(4) Regarding temporal and spatial effects, in terms of regional differences, the western regions are more affected by basic public service equalization policies than the central and eastern regions, and the central regions are more susceptible to such policies than the eastern regions in improving residents' sense of safety. In terms of temporal differences, basic public service equalization policies tend to produce short-term effects that facilitate improvements in residents' sense of safety.

### 7.2 Policy implications

(1) Strengthen the Social Support Mechanism and Advance the Development of a Hierarchical and Region-Specific Social Security System
  We should continuously expand pension insurance coverage, improve the accessibility of the personal pension system to meet the needs and labor force characteristics of different regions, and implement policies to extend pension insurance coverage to vulnerable groups. We will deepen the integrated basic medical insurance system for urban and rural residents to strengthen social support for residents. Efforts should be made to establish a comprehensive promotion mechanism for basic public service equalization policies underpinned by social support, and to continuously optimize its role in improving residents' sense of safety.
(2) Adhere to a Social Equity-Oriented Social Security System and Take Timely, Targeted Measures to Resolve Institutional Disparities
  Given the role of basic public services in enhancing residents' perceptions of social equity and thereby improving their sense of safety, we need to break down household registration and identity-related barriers and advance the construction of a social equity system for migrant populations tailored to national conditions. In line with the hierarchical and time-varying characteristics of policy implementation, we may derive benefits from institutional reforms on a regular or *ad hoc* basis to maintain security and stability in different periods. Meanwhile, we should optimize policies for supply- and demand-side reforms of the social security system within the fiscal framework for low-income groups, ensure social security benefits for all income groups under jurisdiction, and provide hierarchical and distinctive developmental social security content.
(3) Consolidate the Social Trust Mechanism and Enhance Governance Credibility Based on Regional Economic and Cultural Conditions
  Promoting the construction of the social trust mechanism is also a crucial path to improving residents' sense of safety. As this study confirms that social trust serves as a key mediating variable through which basic public service equalization boosts residents' sense of safety, it is necessary to strengthen trust between the government and citizens, given the country's current level of social trust. We should clarify the standards and duration for unemployment insurance benefits based on regional economic conditions, and provide targeted training for unemployed individuals facing difficulties in accordance with regional industrial development needs. By focusing on the preventive functions of social security, we will continuously strengthen the long-term developmental expectation effect of pension and medical insurance on maintaining low unemployment rates.

### 7.3 Discussion

Previous studies have lacked direct, systematic empirical tests linking basic public service equalization, a macrosocial policy, to residents' sense of safety, a comprehensive social mentality. This study addresses this gap by establishing an explicit connection between public services and a sense of safety. Moreover, prior research has failed to systematically examine whether and how these social psychological variables act as key mediating mechanisms within a unified framework that takes public service policies as the starting point; this study fills this research void through mechanism analysis from three dimensions. Additionally, the existing literature has paid insufficient attention to residents' sense of safety across regions in developing countries, and this study complements this deficiency by conducting heterogeneity analyses and discussions across regions and over time in China. The core contribution of this study lies in, for the first time, using nationwide long-term survey data and a quasi-experimental design to empirically test and confirm that basic public service equalization policies exert a significant positive effect on Chinese residents' sense of safety, while systematically revealing the underlying social psychological mechanisms and heterogeneous distribution patterns.

Nevertheless, this study is subject to several limitations, which offer valuable directions for future scholarly inquiry. First, limitations in the research context. The conclusions of this paper are drawn from data under China's specific institutional and cultural background. China's social security reform is characterized by distinct centralized coordination and incremental implementation, and its effects may differ from those in countries with different political systems, welfare models, or development stages. Therefore, the generalizability of the conclusions needs to be assessed and verified across countries.

Second, the prominent sample representativeness bias may undermine the external validity of the conclusions. This study analyses data from the China General Social Survey (CGSS), in which the proportion of the treatment group (individuals covered by the basic public service equalization policy) reaches 88.3%, resulting in an extreme imbalance in sample sizes between the treatment and control groups. Although propensity score matching (PSM) was employed to calibrate the sample and control for differences in observable characteristics between groups, the resulting sample structure still fails to eliminate potential biases entirely. On the one hand, the high proportion of the treatment group may lead to insufficient representativeness of the matched control group, which cannot fully reflect the characteristics of groups not covered by the policy. On the other hand, the unbalanced sample distribution may cause regression results to be more inclined to fit the treatment effect of the treatment group, limiting the inferential ability for non-policy-covered groups and thereby affecting the generalizability of the conclusions across different policy coverage scenarios nationwide.

Third, the mechanism analysis lacks depth, failing to reveal the complex interactions and mediating pathways among variables. This study separately tests the independent mediating effects of social trust, social equity, and social support, confirming their bridging roles in linking basic public service equalization to residents' sense of safety. However, there is a noticeable gap in exploring the intrinsic connections among these mechanisms. From a theoretical perspective, social equity, social trust, and residents' sense of safety do not exist in isolation but may form a chain of mediating paths. The existing analysis overlooks such multi-mechanism linkage relationships and remains at the level of single-mediation testing, failing to fully capture the complex intrinsic logic of policy effect transmission.

Fourth, the measurement of core variables relies on subjective self-reports, which pose risks of bias. Core variables in this study, such as residents' sense of safety, social trust, and social equity, are all measured using subjective evaluation items from the CGSS questionnaire, depending on residents' self-reports and subjective judgments. This measurement method is inevitably affected by factors such as individual cognitive biases, emotional states, and response biases. For instance, some residents may make different evaluations of the same level of public services and social environment due to differences in subjective expectations. Meanwhile, responses to subjective items may be affected by “social desirability bias,” leading to data that do not fully reflect the true situation. Although the study attempts to minimize interference by controlling for individual characteristics, the lack of cross-validation against objective indicators may still affect measurement validity and the robustness of the empirical results. In response to the aforementioned limitations, Future research can undertake more in-depth extensions in the following respects to bolster the scientific rigor and comprehensiveness of the study. First, future work should refine sample design and estimation approaches to enhance the external validity of the findings. On the one hand, the imbalance in sample sizes between the treatment and control groups can be addressed by expanding the sample sources and adopting stratified sampling methods to ensure sufficient representativeness of both groups. On the other hand, a combined model of multi-period difference-in-differences (DID) and PSM can be introduced, and entropy balancing can be used to further calibrate the sample, reducing biases caused by an unbalanced sample structure. In addition, case study methods can be integrated to conduct comparative analyses in regions with different policy coverage rates, verifying the applicability of conclusions across scenarios with different sample structures. Second, construct complex mediation models to reveal chain transmission and interaction effects among mechanisms. Future research can adopt chain mediation models to systematically test potential transmission paths such as “basic public service equalization → social equity → social trust → residents' sense of safety,” clarifying the effect intensity and transmission order of each mechanism variable in the chain. Meanwhile, moderated mediation models can be constructed to analyze the interaction effects among different mechanisms.

Meanwhile, this finding is contingent upon a context of rapid economic growth, strong state capacity for policy implementation, and a specific social contract between the government and citizens. However, China has a unique policy system, accompanied by a strong national execution capability and a relatively rapid economic development background within a specific period, which has presented a rather distinctive trend of changes in residents' sense of security and the evolution characteristics of basic public service guarantee policies. However, its core logic of alleviating social anxiety and enhancing public security through the equalization of basic public services reveals that when the government can effectively perform the redistributive function and provide stable social insurance and public services to citizens, it can significantly reduce individuals' expectations of future uncertainty, thereby enhancing the overall sense of security and cohesion of society. This conclusion provides empirical evidence from a super-large country for policymakers worldwide who are committed to promoting social stability through social investment.

In conclusion, adopt a combined subjective-objective measurement approach to improve the validity of variable measurement. On the one hand, subjective items from the CGSS questionnaire can be integrated with objective indicators. For example, objective data, such as the completeness of community public service facilities and the actual coverage rate of social security policies, can be used to cross-validate residents' subjective evaluations and reduce the impact of subjective biases. On the other hand, experimental or quasi-experimental methods can be used, and small-scale questionnaires or field experiments can be conducted by setting up control and experimental groups to more accurately capture causal relationships among core variables, providing multidimensional support for empirical results. Fourth, establish a multi-policy analytical framework to explore policy synergy effects and heterogeneity.

Future research can incorporate basic public service equalization policies into a unified model alongside related policies, such as employment, social security, and public security. Methods, such as policy interaction terms and policy mix analysis can be used to test the synergetic, complementary, or substitution effects among different policies. Meanwhile, policy heterogeneity analysis can be further refined—for example, by exploring differences in the interaction effects between basic public service equalization policies and different types of employment policies across urban-rural areas and income groups—to provide a more precise basis for policy mix optimisation. In addition, by combining the time nodes of policy implementation, the effects of changes in the dynamic adjustment process of multiple policies can be analyzed to enrich the dynamic analytical perspective on policy effects.

## Data Availability

The raw data supporting the conclusions of this article will be made available by the authors, without undue reservation.
