# Correction: Does the reform of equalization of basic public services affect residents' sense of safety?—A case from China

**DOI:** 10.3389/fpubh.2026.1859789

**Published:** 2026-05-06

**Authors:** Zhengqi Han

**Affiliations:** Policing School, People's Public Security University of China, Beijing, China

**Keywords:** difference-in-differences method, equalization of basic public services, people's well-being, residents' sense of safety, social security reform

Affiliation Policing School, People's Public Security University of China, Beijing, China was erroneously given as School of Humanity and Law, Hebei University, Shijiazhuang, China.

The original version of this article has been updated.

